# Effects of Favorable Alleles for Water-Soluble Carbohydrates at Grain Filling on Grain Weight under Drought and Heat Stresses in Wheat

**DOI:** 10.1371/journal.pone.0102917

**Published:** 2014-07-18

**Authors:** Bin Zhang, Weiyu Li, Xiaoping Chang, Runzhi Li, Ruilian Jing

**Affiliations:** 1 National Key Facility for Crop Gene Resources and Genetic Improvement/Institute of Crop Science, Chinese Academy of Agricultural Sciences, Beijing, China; 2 College of Agronomy, Shanxi Agricultural University, Taigu, China; International Rice Research Institute, Philippines

## Abstract

Drought, heat and other abiotic stresses during grain filling can result in reductions in grain weight. Conserved water-soluble carbohydrates (WSC) at early grain filling play an important role in partial compensation of reduced carbon supply. A diverse population of 262 historical winter wheat accessions was used in the present study. There were significant correlations between 1000-grain weight (TGW) and four types of WSC, viz. (1) total WSC at the mid-grain filling stage (14 days after flowering) produced by leaves and non-leaf organs; (2) WSC contributed by current leaf assimilation during the mid-grain filling; (3) WSC in non-leaf organs at the mid-grain filling, excluding the current leaf assimilation; and (4) WSC used for respiration and remobilization during the mid-grain filling. Association and favorable allele analyses of 209 genome-wide SSR markers and the four types of WSC were conducted using a mixed linear model. Seven novel favorable WSC alleles exhibited positive individual contributions to TGW, which were verified under 16 environments. Dosage effects of pyramided favorable WSC alleles and significantly linear correlations between the number of favorable WSC alleles and TGW were observed. Our results suggested that pyramiding more favorable WSC alleles was effective for improving both WSC and grain weight in future wheat breeding programs.

## Introduction

Wheat (*Triticum aestivum* L.) is one of the most important crops in the world, feeding nearly half the world population [1]. High grain yield is the most important breeding objective in wheat improvement. Drought, heat and other abiotic stresses greatly affect growth and productivity of wheat, especially during grain filling stage. Grain filling in wheat depends on two major sources of carbon: current photosynthate in leaves and non-leaf organs; and carbohydrates stored in the stem and leaf sheath from stem elongation to the early phase of grain filling [2]. The latter can be important in buffering grain yields against unfavorable conditions for photosynthesis during the grain-filling period [3], [4].

Water-soluble carbohydrates (WSC) accumulation and utilization depend on growing conditions and genotypes, and there may be differences between internodes [2], [5], [6]. Among three segments of the main stem (peduncle, penultimate internode and the remainder segments), the remainder segments are the major storage sites and the major source for WSC mobilization during the grain filling period [7]. In general, WSC accumulate until 10–20 days after anthesis, and the reserved WSC can reach more than 40% of total stem dry weight in wheat [8]. The contribution of WSC to final yield and kernel size is 10%–20% of total grain weight under normal condition [9]. Drought stress during grain filling, often involving not only water stress but also heat, inhibits current assimilation and damages photosynthetic organs, especially leaves. When photosynthetic activity is suppressed, the reserved WSC play a more important role in partial compensation of the reduced carbon supply. In addition, drought induced reserved WSC mobilization with higher efficiency, potentially contributing up to 70% of grain dry matter [8], [10].

Based on a large group of genotypes with various WSC contents, the ranking of wheat lines for WSC is consistent across diverse environments. Stem WSC content shows high broad-sense heritability (*h* ^2^ = 0.9). WSC are inversely related to stem number but genotypic ranking persists when compared at similar stem densities [11], [12]. In past years, selection for high WSC in stems occurred during development of drought-tolerant wheat varieties in the UK and Australia [13], [14]. It has been suggested that the release of representative UK wheat cultivars from 1972 to 1995 was associated with increasing stem WSC content [15]. Therefore, high stem WSC content was suggested as a useful trait for improving grain weight in wheat breeding programs [11], [14], [15].

Variation in stem WSC among wheat genotypes is an important genetic factor involving grain weight and yield under drought stress conditions [16]. Thus, knowledge of stem WSC is essential for understanding yield-limiting factors and for improving yield potential in wheat. QTL associated with stem WSC have been reported in perennial ryegrass [17], rice [18], maize [19], barley [20], and wheat [21]–[23]. In wheat, QTL for WSC were mapped on chromosomes 1A, 2D, 4A, 4B, 5D, 6B, 7B and 7D. QTL for drought tolerance also appeared in homologous regions on the group 7 chromosomes [22]. Yang et al. [23] discovered eight, one and two additive QTL for WSC at flowering, grain-filling and maturity, respectively. However, WSC content is a complex quantitative trait controlled by polygenes, and the small effects of many independent QTL limit their direct use for marker-assisted selection in breeding programs [8], [24].

Photosynthesis is the all-important metabolic process determining grain yield in wheat. When water deficit occurs during grain filling stage, photosynthetic rates significantly decrease in leaf blades and non-leaf green organs, such as leaf sheath, glume and awn [25]–[27]. However, non-leaf green organs are relatively more stable than leaf blades [27]. In this study, four types of WSC (Total, Leaf, Non-leaf and Remo) under drought stress (DS) and well-watered (WW) conditions in 262 winter wheat accessions were mainly used, viz. Total, total WSC at the mid-grain filling stage (14 days after flowering) produced by leaves and non-leaf organs; Leaf, WSC contributed by current leaf assimilation during the mid-grain filling; Non-leaf, WSC in non-leaf organs at the mid-grain filling, excluding the current leaf assimilation; Remo, WSC used for respiration and remobilization during the mid-grain filling. The objectives were to (1) detect the relative contributions of leaf and non-leaf organs during grain filling stage to WSC and 1000-grain weight (TGW) under two water regimes; (2) explore genetic resources with high WSC by association analysis; (3) verify the stable favorable WSC alleles with significant effect on TGW under 16 environments (5 drought stress conditions, 3 drought and heat stress (HS) conditions, 5 well-watered conditions, 3 well-watered and heat stress conditions). We observed a significantly positive contribution of WSC to TGW, and the results of favorable WSC allele analysis will be helpful for wheat breeders in selecting genotypes with higher TGW.

## Materials and Methods

### Ethics statement

Two locations, Changping (116°13′E; 40°13′N) and Shunyi (116°56′E; 40°23′N) in Beijing, are the experiment stations of the Institute of Crop Science, Chinese Academy of Agricultural Sciences. We have obtained the relevant permission for our field studies for growing our plant materials in the field from the corresponding institutions. There was no specific permissions required for these locations/activities. Our field studies did not involve endangered or protected species.

### Plant materials and field experiments

A population of 262 common wheat accessions were used in our research [28], [29]; 254 were from China, 3 from USA, 2 from Australia, 2 from Italy, and 1 from Romania (Table S1). The Chinese accessions were mainly from the Northern Winter Wheat Zone, and Yellow and Huai River Valleys Facultative Wheat Zone, including landraces and modern cultivars released from the 1940s to 2000s. All were planted at the beginning of October and harvested in the following mid-June. The experimental unit was a 2 m 4-row plot with 0.3 m between rows. There were 40 plants per row. The field was managed under separate rain-fed (drought stress, DS) and well-watered (WW) conditions. The WW plots were watered with 750 m^3^/ha (75 mm) at the pre-overwintering, booting, flowering and grain filling stages, respectively.

The materials were grown in Changping, Beijing, the experiment station of the Institute of Crop Science in 2010, for collecting data on WSC at the mid-grain filling (14 days after flowering) and TGW at maturity. The treatments involved cutting spikes at flowering stage, removing leaves at flowering stage and a normal control. The rainfall from sowing to harvest was 131 mm. The field was managed under DS and WW conditions.

The population was planted in Changping and Shunyi, Beijing, the experiment stations of the Institute of Crop Science, over 3 years for measuring TGW at maturity. The planting years were 2009 and 2010 at both Changping and Shunyi, and 2011 in Shunyi. The rainfalls in the growing seasons were 192 mm, 131 mm and 180 mm, respectively. The field was also managed under DS and WW conditions. A greenhouse experiment was conducted at Shunyi; at heading polythene covers were placed over selected heads to increase the temperature and thereby simulate heat stress (HS). Thus there were four treatments (DS, WW, DS+HS and WW+HS), with E1 to E16 indicating the environments of Changping in 2009 under DS and WW; Shunyi in 2009 under DS, DS+HS, WW and WW+HS; Changping in 2010 under DS and WW; Shunyi in 2010 under DS, DS+HS, WW and WW+HS; Shunyi in 2011 under DS, DS+HS, WW and WW+HS, respectively.

### Phenotyping of WSC and TGW

The methods of collecting data on WSC were reported earlier [30]. For each genotype, five main stems with the same heading date were selected as samples. The main stem was cut from the soil surface at the mid-grain filling (14 days after flowering). Leaf blades were removed, and stem samples were cut into three parts, the upmost internode (peduncle, Ped), the lower internode (the remainder segments of stem except for peduncle, Low) and the spike. The fresh samples were dehydrated until a constant dry weight. The WSC of the three sections, i.e. peduncle, the lower internode and whole stem (Ste), were determined by different near-infrared reflectance spectroscopy regression models, which were developed for quantitative determination of WSC using samples of 150 doubled haploid lines (Hanxuan 10×Lumai 14) [30]. Briefly, as the first step, partial least square regression models for predicting WSC in the target parts of wheat were developed using selected wavelength regions, spectroscopy pretreatments and latent variables included in each model. The amounts of WSC (mg WSC/g dry weight, mg/g dw) in each modeling sample of 150 doubled haploid lines were also measured by chemical assay (anthrone colorimetric assay), and used for cross validation. WSC were extracted according to the modified procedure described by Wardlaw and Willenbrink [2], [30]; the amounts of WSC were measured as fructose equivalents using the anthrone colorimetric assay at 620 nm by 722S spectrophotometer [31]. This showed that the near-infrared reflectance spectroscopy regression models were highly accurate in determining the true values of WSC measured by chemical assay in the wheat organs tested (coefficient of determination *R* ^2^>0.992 and root mean square error of prediction RMSEP<0.228). In addition, 40 samples per model (i.e. not included in the modeling samples) were used to assess the models. The results confirmed the high quality of the models in evaluating WSC.

We obtained four types of WSC (Total, Leaf, Non-leaf, and Remo), viz. Total, the total WSC at the mid-grain filling produced by leaves and non-leaf organs which was obtained from the treatment of cutting spikes (WSC_cutting spikes_); Leaf, WSC contributed by current leaf assimilation during the mid-grain filling, i.e. the reduction in WSC due to cutting leaves which was estimated by comparing WSC between the normal control and the treatment of cutting leaves (WSC_untreated_ – WSC_removing leaves_); Non-leaf, the WSC in non-leaf organs at the mid-grain filling (excluding the current leaf assimilation) which was estimated by the treatment of removing leaves (WSC_removing leaves_); Remo, WSC used for respiration and remobilization during the mid-grain filling which was obtained by comparing WSC between the normal control and the treatment of cutting spikes (WSC_cutting spikes_ – WSC_untreated_).

Spikes corresponding to main stem samples were collected at maturity stage for each accession to obtain TGW. The reduction of TGW due to leaf removal was calculated for each cultivar as: [(TGW_untreated_ – TGW_removing leaves_)/TGW_untreated_]×100%.

### SSR genotyping and association mapping

Two hundred and nine SSR markers, evenly distributed on the 21 wheat chromosomes, were selected for evaluating population structure, relative kinship, and association mapping. The genetic positions of SSR markers were obtained from the consensus map Ta-SSR-2004 [32] and the Komugi wheat genetic resources database (http://www.shigen.nig.ac.jp/wheat/komugi?/top/top.jsp). Fluorescent primers were synthesized by ABI (Applied Biosystems, Foster City, CA, USA). Amplification products were separated on an ABI3730 DNA Analyzer, and fragment sizes were analyzed by GeneMapper software (Applied Biosystems).

Allele number, allele frequency and polymorphism information content were calculated by PowerMarker V3.25 [33]. Population structure was estimated by STRUCTURE v2.3.2 using data from 209 SSR markers. The number of hypothetical subpopulations was set from *k* = 1 to 10 with a burn-in period of 50,000 iterations and a run of 500,000 replications of Markov Chain Monte Carlo after burn in. The △*k* method was applied according to LnP(D) in STRUCTURE [34]. The *Q* data of five replicate runs were integrated by CLUMPP software [35]. Principal coordinate analysis based on genetic distances was also used to confirm the results of STRUCTURE by NTSYSpc analysis [36]. The relative kinship coefficient (*K*) was calculated by the SPAGeDi software package [37]. Finally, the *Q*+*K* models were performed using mixed linear model in TASSEL V2.1 for association of SWSC [38], [39].

## Results

### Contribution of leaf and non-leaf organs to 1000-grain weight during grain filling

Figure S1 summarizes the relative contributions of leaf and non-leaf organs to final 1000-grain weight during grain filling across the 262 diverse winter wheat genotypes. Reduction in TGW due to cutting leaves (i.e. the contribution of leaves to TGW during grain filling) was 14.79% (6.26 g) at maturity under DS, compared to 19.84% (8.56 g) under WW condition (Figure S1). The lower contribution of leaves to TGW under DS condition reflected the negative effect of water deficit on photosynthetic rates in leaf blades during grain filling. Non-leaf organs contributed 85.21% (36.05 g) to TGW under DS, whereas it was 80.16% (34.60 g) under WW condition (Figure S1).

### Variation in WSC of leaves and non-leaf organs in different internodes at the mid-grain filling under two water regimes

**Figure 1:**
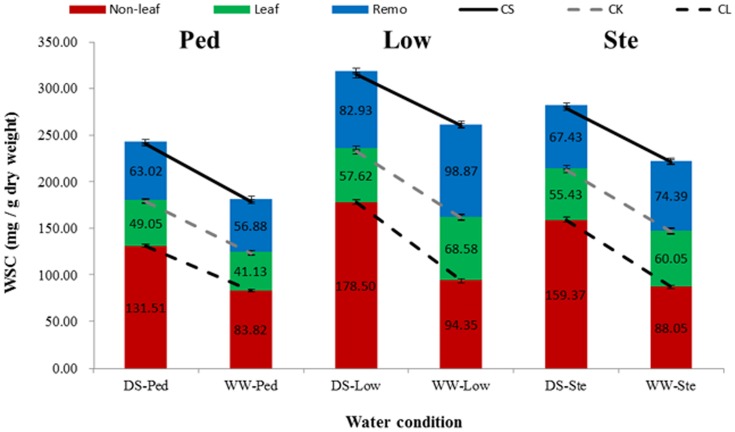
WSC (mg/g dw) of different internodes at the mid-grain filling stage (14 days after flowering) under well-watered and drought stress conditions. Bars indicate 2SE. WSC, water-soluble carbohydrates; DS-Ped, peduncle under drought stress; WW-Ped, peduncle, well-watered; DS-Low, lower internode, drought stress; WW-Low, lower internode, well-watered; DS-Ste, whole stem, drought stress; WW-Ste, whole stem, well-watered; CS, cutting spikes; CL, removing leaves; CK, normal control; Non-leaf, WSC in non-leaf organs at the mid-grain filling, excluding the current leaf assimilation; Leaf, WSC contributed by current leaf assimilation during the mid-grain filling; Remo, WSC used for respiration and remobilization during the mid-grain filling.

The WSC in lower internodes (the remainder segments of stem except for peduncle, Low) were higher than those in peduncles (the upmost internode, Ped; Figure 1) under both water regimes in all types of WSC. The WSC in non-leaf organs at the mid-grain filling ranged from 83.82 to 178.50 mg WSC/g dry weight (mg/g dw), and those contributed by current leaf assimilation were from 41.13 to 68.58 mg/g dw, thus showing the relative importance of stem-reserved WSC for grain filling. The WSC used for Remo at the mid-grain filling ranged from 56.88 to 98.87 mg/g dw (Figure 1). WSC in non-leaf organs were 131.51, 178.50 and 159.37 mg/g dw in peduncles, lower internodes and the whole stem under drought stress, and 83.82, 94.35 and 88.05 mg/g dw under well-watered condition, respectively; the ratios between two water regimes were 156.90%, 189.19% and 181.00%, respectively (Figure 1). This implied that long term drought stress triggered a series of metabolic reactions by increasing fructans for self-protection. At the mid-grain filling, WSC contributed by current leaf assimilation were 57.62 and 55.43 mg/g dw in the lower internode and whole stem under drought stress, compared with 68.58 and 60.05 mg/g dw under well-watered condition (Figure 1). Thus drought during grain filling greatly influences current photosynthesis and dry matter accumulation.

### Correlations between WSC at the mid-grain filling and TGW

WSC are recognized as an important source of grain dry matter for grain filling in wheat. There were significant correlations between the four types of WSC (Total, Non-leaf, Leaf and Remo) at the mid-grain filling and TGW (Table 1, Table S2∼S3). Moreover, there were higher correlations between the four types of WSC and TGW under drought stress compared to those under well-watered condition (Table 1). Under drought stress, WSC of Total was significantly correlated with TGW (*r* = 0.248^***^, 0.386^***^ and 0.392^***^); and correlations between WSC of Non-leaf, Leaf and TGW were *r* = 0.140^*^ to 0.275^***^, 0.156^*^ to 0.220^***^, respectively. Under well-watered condition, there were three instances of significant correlations between WSC of Total and TGW (*r* = 0.135^*^, 0.146^*^ and 0.176^**^).

**Table 1:** Pearson correlation coefficients of WSC at the mid-grain filling and TGW under well-watered and drought stress conditions.

WSC		TGW	
Types	Internodes	DS	WW
Total^a^	Ped	0.248***	0.135*
	Low	0.386***	0.146*
	Ste	0.392***	0.176**
Leaf^a^	Ped	0.218***	0.027
	Low	0.156*	0.100
	Ste	0.220***	0.071
Non-leaf^a^	Ped	0.011	−0.046
	Low	0.177**	−0.000
	Ste	0.140*	−0.011
Non-leaf^b^	Ped	0.207***	0.121
	Low	0.275***	0.011
	Ste	0.274***	−0.001
Remo^a^	Ped	−0.014	0.100
	Low	0.106	0.044
	Ste	0.037	0.105

*Significant at *P = *0.05; **Significant at *P = *0.01; ***Significant at *P = *0.001. Total, total WSC at the mid-grain filling produced by leaves and non-leaf organs; Leaf, WSC contributed by current leaf assimilation during the mid-grain filling; Non-leaf, WSC in non-leaf organs at the mid-grain filling, excluding the current leaf assimilation; Remo, WSC used for respiration and remobilization during the mid-grain filling; Ped, peduncle; Low, lower internode; Ste, whole stem; TGW, 1000-grain weight at maturity.

a TGW was measured on the normal control;

b TGW was measured with treatment with removing leaves.

### Association analysis for WSC at the mid-grain filling and the search for favorable alleles

Based on the population structure assessment using 209 markers, the 262 wheat accessions were separated into two sub-populations, comprising 126 and 136 accessions (our unpublished data). Association analysis using the 209 SSR markers and four types of WSC at the mid-grain filling was conducted using a mixed linear model, which accounted for population structure (*Q*) and relative kinship (*K* matrix). Thirteen, 13, 23 and 14 novel loci were significantly (*P*<0.01) associated with WSC of Total, Leaf, Non-leaf and Remo in 17, 17, 31 and 18 instances, respectively (Table S4∼S7). Variances explained by SSR markers (*R* ^2^) ranged from 0.11% to 10.51%. Twenty-two loci were identified more than once. *Xcfd17-2D* (associated with WSC of Remo under WW condition; Remo, WW), *Xgwm513-4B* (Non-leaf, DS) and *Xwmc517-7B* (Non-leaf, WW) were detected in all internodes (peduncle, lower internode and the whole stem). *Xbarc228-2D* (Total, DS), *Xgwm169-6A* (Remo, WW) and *Xgwm537-7B* (Leaf, DS) were detected in both peduncle and lower internode. *Xcfd53-2D* (Non-leaf, DS) and *Xcfa2240-7A* (Non-leaf, DS) were identified in both the peduncle and whole stem; *Xgwm630* (Remo, WW), *Xgwm610-4A* (Leaf, WW), *Xgwm165.1-4D* (Non-leaf, WW) and *Xgwm182-5D* (Total, WW) were similarly identified in both the lower internode and whole stem. *Xbarc125-3D* was associated with WSC of Total in lower internode under both DS and WW conditions. *Xgwm66*, *Xgwm88*, *Xgwm192*, *Xwmc470-2D*, *Xgwm181-3B*, *Xgwm358-5D*, *Xgwm583-5D* and *Xgwm428-7D* were associated with more than one types of WSC (Total, Non-leaf, Leaf and Remo).

For associated loci, we explored favorable WSC alleles by assessing differences in WSC between accessions carrying favorable alleles and those with other alleles using ANOVA (SAS 8.01), i.e. the WSC of the former were significantly (*P*<0.05) higher than those of the latter. There were 7, 10, 12 and 9 novel favorable alleles for WSC of Total, Leaf, Non-leaf and Remo, respectively (Tables S4∼S7). *Xcfd17-2D* (Remo, WW) had the same favorable WSC alleles (*Xcfd17-2D_223_*) in peduncle, lower internode and the whole stem estimates, i.e. 74.1 compared with 51.7 mg/g dw (*P*<0.001), 113.5 compared with 94.3 mg/g dw (*P*<0.05), and 89.7 compared with 69.7 mg/g dw (*P*<0.01), respectively. *Xgwm181-3B_131 and 161_* (Leaf, DS), *Xgwm610-4A_167_* (Leaf, WW), *Xgwm513-4B_144_* (Leaf, DS), *Xgwm165.1-4D_199_* (Non-leaf, WW), *Xwmc517-7B_188_* (Non-leaf, WW) had positive effects both in lower internode and the whole stem. Higher WSC were associated with *Xgwm169-6A_203_* (Remo, WW) and *Xgwm537-7B_205_* (Leaf, DS) in both the peduncle and lower internode. *Xbarc125-3D_147_* (Total) contributed to higher WSC in lower internodes, not only under well-watered conditions but also under drought stress. Some associated loci, however, had various favorable alleles for different types of WSC; for example, accessions carrying the allele *Xgwm513-4B_144_* exhibited higher WSC of Leaf, whereas accessions with the 142 bp allele had higher WSC of Non-leaf in lower internodes under drought-stress conditions.

### Seven novel favorable WSC alleles individually exhibited positive contributions to TGW under well-watered, drought and heat stress conditions

**Figure 2:**
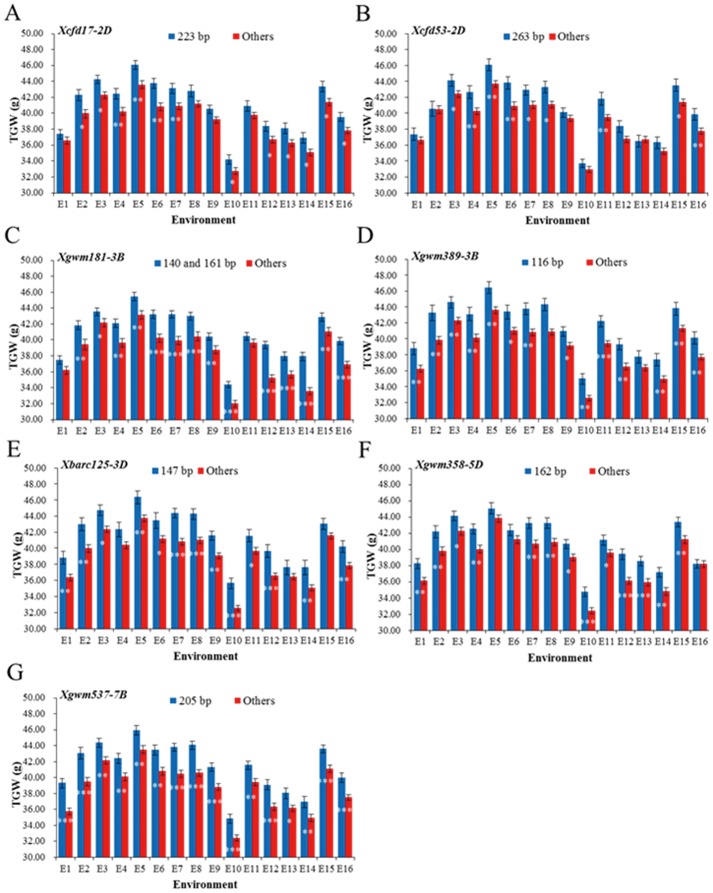
Verification of phenotypic effects of seven novel favorable WSC alleles individually contributing to TGW in sixteen environments. E1, E3, E7, E9 and E13 were drought stress conditions, E6, E12 and E16 were well-watered and heat stress conditions, E4, E10 and E14 were drought and heat stress conditions, E2, E5, E8, E11 and E15 were well-watered conditions. Bars indicate 2SE. *, **, *** Significant at *P* = 0.05, 0.01 and 0.001, respectively.

In order to evaluate the genetic relationship between WSC and TGW, we analyzed the effects of favorable WSC alleles on final TGW by comparing differences in TGW between accessions carrying favorable WSC alleles and those with other alleles. Seven novel favorable WSC alleles exhibited significantly (*P*<0.05) positive contributions to TGW on an individual basis. They were *Xcfd17-2D_223_*, *Xcfd53-2D_263_*, *Xgwm181-3B_140_*_ and *161*_, *Xgwm389-3B_116_*, *Xbarc125-3D_147_*, *Xgwm358-5D_162_* and *Xgwm537-7B_205_* (Table 2). For *Xbarc125-3D_147_*, the higher WSC of Total (341.5 compared to 309.9 mg/g dw) in lower internodes led to a higher TGW (44.99 g compared to 41.14 g) under drought stress conditions; likewise, accessions with this allele also produced higher TGW (43.91 g) than accessions with other alleles (41.85 g) under well-watered conditions, with WSC of 284.3 and 255.9 mg/g dw, respectively. In order to verify the positive contributions of these seven favorable alleles to TGW, we used the same population (262 winter wheat accessions) planted in 16 environments (year×site×water and heat regime combinations; 5 drought stress conditions, 3 well-watered and heat stress conditions, 3 drought and heat stress conditions, 5 well-watered conditions) to confirm the above results. The average TGW of accessions carrying favorable WSC alleles were higher than those without the favorable alleles in all environments (Figure 2).

**Table 2:** Seven favorable WSC alleles individually contributed to significantly higher TGW.

Locus	Trait	Favorable allele (bp)	Freq. (%)	WSC Mean ± SE (mg/g dw)	*P* value	TGW-DS Mean ± SE (g)	*P* value	TGW-WW Mean ± SE (g)	*P* value
*Xbarc125-3D*	Total-Low-DS	147	16.41	341.5±6.0	0***	44.99±0.61	0.0002***	43.91±0.62	0.0488*
		Others	83.59	309.9±3.3		41.14±0.43		41.85±0.44	
	Total-Low-WW	147		284.3±5.5	0***				
		Others		255.9±2.5					
*Xgwm537-7B*	Leaf-Ped-DS	205	28.63	58.0±3.5	0.0039**	43.53±0.61	0.0032**	43.81±0.60	0.0067**
		Others	71.37	45.4±2.4		41.06±0.46		41.52±0.48	
	Leaf-Low-DS	205		65.8±4.3	0.0345*				
		Others		54.2±3.0					
*Xgwm358-5D*	Leaf-Ste-DS	162	30.15	65.4±3.8	0.0064**	43.43±0.72	0.0039**	43.94±0.72	0.0026**
		Others	69.85	51.6±2.7		41.05±0.44		41.43±0.45	
*Xcfd53-2D*	Non-leaf-Ste-DS	263	20.61	168.0±3.4	0.0163*	43.53±0.75	0.0168*	43.80±0.76	0.0326*
		Others	79.39	157.1±2.1		41.30±0.43		41.77±0.44	
*Xgwm181-3B*	Non-leaf-Ste-DS	140 and 161	45.42	163.6±2.6	0.0377*	43.45±0.48	0***	42.91±0.52	0.0834
			54.58	155.9±2.5		40.37±0.54		41.58±0.55	
*Xgwm389-3B*	Remo-Ste-DS	116	19.08	79.4±5.6	0.0152*	44.10±0.74	0.0026**	43.95±0.88	0.0246*
		Others	80.92	64.5±2.7		41.21±0.43		41.76±0.42	
*Xcfd17-2D*	Remo-Ped-WW	223	24.81	74.1±5.1	0.0002***	43.31±0.63	0.0183*	43.75±0.67	0.0193*
		Others	75.19	51.7±2.8		41.25±0.46		41.67±0.46	
	Remo-Low-WW	223		113.5±7.1	0.0123*				
		Others		94.3±3.6					
	Remo-Ste-WW	223		89.7±6.5	0.0044**				
		Others		69.7±3.3					

*Significant at *P = *0.05; **Significant at *P = *0.01; ***Significant at *P = *0.001. Total, total WSC at the mid-grain filling produced by leaves and non-leaf organs; Leaf, WSC contributed by current leaf assimilation during the mid-grain filling; Non-leaf, WSC in non-leaf organs at the mid-grain filling, excluding the current leaf assimilation; Remo, WSC used for respiration and remobilization during the mid-grain filling; Ped, peduncle; Low, lower internode; Ste, whole stem; DS, drought stress; WW, well-watered; TGW, 1000-grain weight at maturity.

### Pyramiding of favorable WSC alleles indicated potential application in wheat breeding

**Figure 3:**
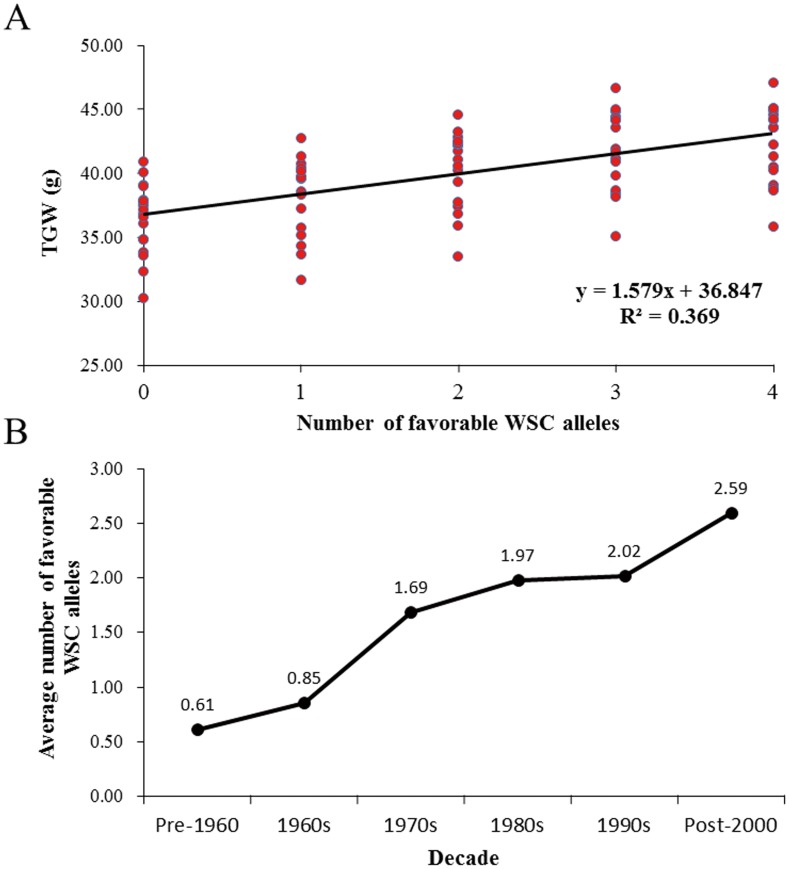
Linear regressions of TGW based on seven favorable WSC alleles in sixteen environments (A), and accumulation of seven favorable WSC alleles in modern varieties released in different decades (B). There were 18, 27, 54, 39, 58 and 51 accessions released in Pre-1960, 1960s, 1970s, 1980s, 1990s and Post-2000, respectively. 15 accessions with unknown released decades were excluded.

To explore whether the pyramiding of favorable WSC alleles showed additive effects, we also analyzed the mean TGW of accessions with different numbers of favorable WSC alleles in 16 environments (Table 3). The average TGW of genotypes with single favorable WSC allele were 31.75 - 42.82 g; TGW with two favorable alleles were 33.57 - 44.66 g; TGW with three ones were 35.17 - 46.76 g; TGW with more than four favorable alleles were 35.90 - 47.18 g across 16 environments. The average TGW of accessions without favorable WSC allele ranged from 30.30 to 41.01 g. In addition, a significantly linear correlation (y = 1.579x+36.847, *R* ^2^ = 0.369) between TGW and number of favorable WSC alleles further confirmed the additive effect (Figure 3A). We also evaluated the distribution of combined favorable WSC alleles in modern varieties from different decades (Figure 3B). The average number of favorable WSC alleles was 0.61 before 1960, and the current average number was 2.59. The increasing number over time reveals a genomic footprint left by breeders, but the relatively low number of 2.59 alleles in current cultivars (post-2000) indicates a potential for pyramiding more favorable alleles [40].

**Table 3:** Pyramiding of favorable WSC alleles contributing to TGW in sixteen environments.

Env.	No. of alleles	TGW^1^ Mean ± SE (%)	Fre. (%)	Env.	No of alleles	TGW^1^ Mean ± SE (%)	Fre. (%)
E1	≥4	39.17±0.75 (A)	13.73	E9	≥4	41.38±0.65 (A)	13.46
(DS)	3	38.47±0.92 (A)	16.08	(DS)	3	41.25±0.57 (A)	16.92
	2	37.48±0.62 (AB)	27.06		2	40.34±0.54 (AB)	26.54
	1	35.79±0.67 (BC)	23.14		1	38.62±0.72 (B)	23.08
	0	33.94±0.81 (C)	20.00		0	36.69±0.71 (C)	20.00
E2	≥4	43.67±1.09 (A)	12.94	E10	≥4	35.90±0.67 (A)	13.46
(WW)	3	42.01±0.80 (AB)	16.86	(DS+HS)	3	35.17±0.62 (AB)	16.92
	2	41.82±0.70 (AB)	27.06		2	33.57±0.54 (B)	26.54
	1	39.72±0.92 (B)	23.92		1	31.75±0.61 (C)	23.08
	0	36.17±1.04 (C)	19.22		0	30.30±0.67 (C)	20.00
E3	≥4	45.17±0.70 (A)	13.41	E11	≥4	42.35±0.83 (A)	13.51
(DS)	3	45.10±0.61 (A)	16.48	(WW)	3	41.77±0.71 (A)	16.60
	2	43.33±0.62 (AB)	26.82		2	40.68±0.56 (A)	26.64
	1	41.40±0.76 (BC)	23.37		1	38.43±0.79 (B)	23.17
	0	40.16±0.81 (C)	19.92		0	37.91±0.68 (B)	20.08
E4	≥4	43.66±0.89 (A)	13.13	E12	≥4	40.54±0.68 (A)	13.46
(DS+HS)	3	43.67±0.70 (A)	16.99	(WW+HS)	3	40.99±0.64 (A)	16.92
	2	41.15±0.67 (B)	26.25		2	37.81±0.63 (B)	26.54
	1	39.82±0.80 (B)	23.55		1	34.38±0.69 (C)	23.08
	0	36.95±0.77 (C)	20.08		0	33.69±0.61 (C)	20.00
E5	≥4	47.18±0.82 (A)	13.03	E13	≥4	38.93±0.95 (A)	13.62
(WW)	3	46.76±0.63 (AB)	16.86	(DS)	3	38.71±0.71 (A)	16.73
	2	44.66±0.63 (BC)	26.82		2	36.89±0.67 (AB)	26.46
	1	42.82±0.83 (CD)	23.37		1	35.22±0.68 (B)	22.96
	0	41.01±0.82 (D)	19.92		0	34.91±0.73 (B)	20.23
E6	≥4	44.21±1.00 (A)	12.85	E14	≥4	38.77±0.93 (A)	13.62
(WW+HS)	3	44.44±0.71 (A)	16.06	(DS+HS)	3	38.28±0.71 (A)	16.73
	2	42.49±0.70 (AB)	26.91		2	35.98±0.68 (B)	26.46
	1	40.45±0.81 (B)	24.10		1	33.72±0.69 (C)	22.96
	0	37.55±0.85 (C)	20.08		0	32.37±0.62 (C)	20.23
E7	≥4	45.04±0.70 (A)	13.46	E15	≥4	44.32±0.78 (A)	13.67
(DS)	3	44.41±0.61 (A)	16.92	(WW)	3	44.26±0.67 (A)	16.80
	2	42.36±0.59 (B)	26.54		2	42.57±0.63 (A)	26.17
	1	39.68±0.74 (C)	23.08		1	40.21±0.70 (B)	23.05
	0	37.22±0.82 (D)	20.00		0	39.08±0.79 (B)	20.31
E8	≥4	45.15±0.85 (A)	13.46	E16	≥4	40.36±0.84 (A)	13.57
(WW)	3	44.24±0.68 (AB)	16.92	(WW+HS)	3	39.88±0.55 (A)	16.67
	2	42.40±0.57 (B)	26.54		2	39.42±0.61 (A)	26.36
	1	39.72±0.78 (C)	23.08		1	37.36±0.68 (B)	23.26
	0	37.96±0.74 (C)	20.00		0	34.86±0.60 (C)	20.16

1 Values with different letters are significantly different (*P*<0.05). DS, drought stress; WW+HS, well-watered and heat stress; DS+HS, drought and heat stress; WW, well-watered.

## Discussion

### Consistency between WSC and TGW under stress conditions

Previous studies suggested that increases in grain yield can mainly be attributed to better partitioning of photosynthetic products [41]. WSC accumulation ability and its remobilization efficiency are much higher in the internodes of drought tolerant cultivars than those of sensitive genotypes under both normal and stress conditions. On the other hand, fructans, the major components of WSC, insert between the head groups of phospholipids, acting as compatible solutes in cells to protect cell membranes and proteins from osmotic damage [42], [43]. Stem samples of rainfed wheat have significantly higher average fructan than irrigated samples. In our research four types of WSC (Total, Leaf, Non-leaf and Remo) under drought stress were overall higher than those under well-watered condition (Figure 1). It has been reported that fructan synthesis is induced by drought stress, and that drought tolerant plants can manufacture more fructans. The tolerant cultivars activate their protection mechanisms faster and more efficiently than the sensitive ones to cope with stress conditions [10], [44].

Drought stress during grain filling can result in reductions in grain weight, due to lower numbers of endosperm cells and a limited maximum storage capacity of the kernels [45], [46]. WSC are recognized as an important source of grain dry matter for grain filling, especially when current photosynthesis is inhibited by drought stress. Water deficit during grain filling stimulates senescence of the whole plant and enhances remobilization of reserved WSC to the grains [47], [48]. Thus, the reserved WSC assimilated pre-anthesis and current assimilation are critically important for grain filling. In the present study, we observed that final grain yield mainly depends on pre-anthesis assimilation by green organs and current photosynthesis of non-leaf organs during grain filling, especially under drought stress condition (Figure S1). In addition, compared with those under well-watered condition, higher correlations between the four types of WSC at the mid-grain filling and TGW under drought stress indicate that yield in unfavorable conditions relies more on pre-stored carbohydrates (Table 1). Association and favorable allele analyses were conducted on four types of WSC (Total, Leaf, Non-leaf and Remo) at the mid-grain filling. Seven novel favorable WSC alleles made positive individual contributions to final TGW under well-watered, drought and heat stress conditions (Table 2, Figure 2).

### Complex relationship between WSC and TGW

WSC accumulation and remobilization are influenced by many factors, making the relationship between WSC and TGW more complex. For example, WSC remobilization is affected by N fertilizers and water deficit [6], [49]. Heavy use of N fertilizers delays plant senescence and reduces the remobilization of prestored assimilates; whereas water deficit performs the reverse function. Thus final TGW is often significantly increased with heavy use of N fertilizers under drought stress, even higher than that under well-watered conditions [49], [50]. WSC in the stem are negatively correlated with tiller per unit area, that is, WSC accumulation is dependent on plant density [8], [12]. Moreover, WSC accumulation is also affected by stem length (plant height) and stem weight. We calculated Pearson correlation coefficients between the four types of WSC at the mid-grain filling and TGW, and we also evaluated the effects of favorable WSC alleles on TGW to further understand their relationships at the genetic level. The results showed that (1) the correlations between them were significant but not robust, the highest Pearson correlation being only *r* = 0.393^***^, and there was no relationship between WSC of Remo at the mid-grain filling and TGW; and (2) there were 7, 10, 12 and 9 favorable alleles for WSC of Total, Leaf, Non-leaf and Remo, respectively. However, only seven favorable WSC alleles exhibited positive individual contributions to TGW. The complex relationship between WSC and TGW due to many influential factors may help us to understand the reasons for these results. In addition, cutting spikes or removing leaves at flowering change the source-sink relationship during grain filling and therefore the four types of WSC at the mid-grain filling might not fully reflect the situation under normal condition.

### Seven favorable WSC alleles will help to improve breeding progress in yield potential

Broad-sense heritability of WSC is relatively high, but shows wide fluctuations under different conditions, i.e. WSC are very sensitive to environments [11], [23]. Yang et al. [23] reported that (1) QTL for WSC accumulation and remobilization could have different expression patterns at different growth stages or in different environments; and (2) 7 of 10 significantly additive QTL for WSC interacted with environment. Thus, stable molecular markers for WSC are essential to understand its genetic basis. Moreover, exploration of favorable WSC alleles in germplasm resources could be useful to plant breeders, but the effectiveness of such alleles needs to be verified [29], [51]. In this study, seven favorable WSC alleles significantly (*P*<0.05) enhanced TGW on an individual basis (Table 2). An additive QTL for WSC, *QSwscf.cgb-2D.1* (*WMC453.1*–*WMC18*), was detected in a Hanxuan 10×Lumai 14 doubled haploid population [23]. *Xcfd17-2D* was 1.5 cM from the flanking marker *Xwmc18-2D*. *QReswc.cgb-3B*, controlled WSC and remobilization efficiency, and *Xgwm181-3B* shares one of its flanking markers (*Xgwm547*–*Xgwm181*). Adjacent chromosome intervals, such as *QAeswc.cgb-3B.1*, *QSwscm.cgb-3B.1* and *QSwscf.cgb-3B*, carry QTL for WSC and its accumulation efficiency [23]. In addition, *Xcfd53-2D*, *Xgwm389-3B* and *Xgwm537-7B* were associated with yield-related traits [49]–[54]. *Xbarc125-3D* was also associated with TGW (our unpublished data).

The seven favorable WSC alleles for enhancing TGW were verified under 16 environments (5 drought stress conditions, 3 well-watered and heat stress conditions, 3 drought and heat stress conditions, and 5 well-watered conditions) using a population of 262 winter wheat accessions (Table 2 and Figure 2). Many studies show that marker-based strategies of gene pyramiding are effective [27], [55], [56]. A dosage effect of pyramiding seven favorable WSC alleles (Table 3, Figure 3A) was also demonstrated in the study. The accumulation of favorable WSC alleles over different decades also indicated that they had been individually selected by breeders in the past and that there is potential for further improvement in the future.

## Supporting Information

Figure S1 **The percentage contributions of leaf and non-leaf organs to 1000-grain weight (TGW) under drought stress (DS) and well-watered (WW) conditions during grain filling.** Bars indicate 2SE. The data in the columns were the absolute values of TGW (g).
(TIF)

Table S1 **262 common wheat accessions and their origins.**
(XLSX)

Table S2 **Statistic data of WSC (mg/g dw) at the mid-grain filling under well-watered and drought stress conditions.**
(XLSX)

Table S3 **Statistic data of TGW under well-watered and drought stress conditions.**
(XLSX)

Table S4 **Thirteen loci significantly associated with the total WSC at the mid-grain filling produced by leaves and non-leaf organs (Total) and phenotypic values of favorable marker alleles under two water regimes.**
(XLSX)

Table S5 **Thirteen loci significantly associated with the WSC contributed by current leaf assimilation during the mid-grain filling (Leaf) and phenotypic values of favorable marker alleles under two water regimes.**
(XLSX)

Table S6 **Twenty-three loci significantly associated with the WSC in non-leaf organs at the mid-grain filling (excluding the current leaf assimilation, Non-leaf) and phenotypic values of favorable marker alleles under two water regimes.**
(XLSX)

Table S7 **Fourteen loci significantly associated with the WSC used for respiration and remobilization during the mid-grain filling (Remo) and phenotypic values of favorable marker alleles under two water regimes.**
(XLSX)

## References

[1] Gupta P, Mir R, Mohan A, Kumar J (2008) Wheat genomics: present status and future prospects. Int J Plant Genomics doi:10.1155/2008/896451 10.1155/2008/896451 PMC2397558 18528518

[2] Wardlaw IF, Willenbrink J (1994) Carbohydrate storage and mobilisation by the culm of wheat between heading and grain maturity: the relation to sucrose synthase and sucrose-phosphate synthase. Funct Plant Biol 21: 255–271.

[3] Gallagher J, Biscoe P, Hunter B (1976) Effects of drought on grain growth. Nature 264: 541–542.

[4] Bidinger F, Musgrave R, Fischer R (1977) Contribution of stored pre-anthesis assimilate to grain yield in wheat and barley. Nature 270: 431–433.

[5] Spollen WG, Nelson CJ (1994) Response of fructan to water deficit in growing leaves of tall fescue. Plant Physiol 106: 329–336. 12232332 10.1104/pp.106.1.329 PMC159531

[6] Blum A (1998) Improving wheat grain filling under stress by stem reserve mobilisation. Euphytica 100: 77–83.

[7] Ehdaie B, Madore M, Waines J, Alloush G (2006) Genotypic variation for stem reserves and mobilization in wheat: II. Postanthesis changes in internode water-soluble carbohydrates. Crop Sci 46: 2093–2103.

[8] Rebetzke G, Van Herwaarden A, Jenkins C, Weiss M, Lewis D, et al (2008) Quantitative trait loci for water-soluble carbohydrates and associations with agronomic traits in wheat. Crop and Pasture Sci 59: 891–905.

[9] Gebbing T, Schnyder H (1999) Pre-anthesis reserve utilization for protein and carbohydrate synthesis in grains of wheat. Plant Physiol 121: 871–878. 10557235 10.1104/pp.121.3.871 PMC59449

[10] Goggin DE, Setter TL (2004) Fructosyltransferase activity and fructan accumulation during development in wheat exposed to terminal drought. Funct Plant Biol 31: 11–21. 10.1071/FP03123 32688876

[11] Ruuska SA, Rebetzke GJ, van Herwaarden AF, Richards RA, Fettell NA, et al (2006) Genotypic variation in water-soluble carbohydrate accumulation in wheat. Funct Plant Biol 33: 799–809. 10.1071/FP06062 32689291

[12] Dreccer MF, Chapman SC, Rattey AR, Neal J, Song Y, et al (2013) Developmental and growth controls of tillering and water-soluble carbohydrate accumulation in contrasting wheat (*Triticum aestivum* L.) genotypes: can we dissect them? J Exp Bot 64: 143–160. 23213136 10.1093/jxb/ers317 PMC3528026

[13] Foulkes M, Scott R, Sylvester-Bradley R (2002) The ability of wheat cultivars to withstand drought in UK conditions: formation of grain yield. J Agric Sci 138: 153–169.

[14] van Herwaarden AF, Richards RA (2002) Water-soluble carbohydrate accumulation in stems is related to breeding progress in Australian wheats. In: Plant Breeding for the 11th Millenium, JAMcComb (ed), Proceedings 12th Australasian Plant Breeding Conference (Perth, 15–20 September 2002): 878–882.

[15] Shearman V, Sylvester-Bradley R, Scott R, Foulkes M (2005) Physiological processes associated with wheat yield progress in the UK. Crop Sci 45: 175–185.

[16] Xue GP, McIntyre CL, Jenkins CLD, Glassop D, Van Herwaarden AF, et al (2008) Molecular dissection of variation in carbohydrate metabolism related to water-soluble carbohydrate accumulation in stems of wheat. Plant Physiol 146: 441–454. 18083795 10.1104/pp.107.113076 PMC2245852

[17] Turner LB, Cairns AJ, Armstead IP, Ashton J, Skot K, et al (2006) Dissecting the regulation of fructan metabolism in perennial ryegrass (*Lolium perenne* L.) with quantitative trait locus mapping. New Phytol 169: 45–57. 16390418 10.1111/j.1469-8137.2005.01575.x

[18] Nagata K, Shimizu H, Terao T (2002) Quantitative trait loci for nonstructural carbohydrate accumulation in leaf sheaths and culms of rice (*Oryza sativa* L.) and their effects on grain filling. Breed Sci 52: 275–283.

[19] Thevenot C, Simond-Cote E, Reyss A, Manicacci D, Trouverie J, et al (2005) QTLs for enzyme activities and soluble carbohydrates involved in starch accumulation during grain filling in maize. J Exp Bot 56: 945–958. 15710637 10.1093/jxb/eri087

[20] Teulat B, Borries C, This D (2001) New QTLs identified for plant water status, water-soluble carbohydrate and osmotic adjustment in a barley population grown in a growth-chamber under two water regimes. Theor Appl Genet 103: 161–170.

[21] Galiba G, Kerepesi I, Snape J, Sutka J (1997) Location of a gene regulating cold-induced carbohydrate production on chromosome 5A of wheat. Theor Appl Genet 95: 265–270.

[22] Salem KFM, Roder MS, Borner A (2007) Identification and mapping quantitative trait loci for stem reserve mobilisation in wheat (*Triticum aestivum* L.). Cereal Res Commun 35: 1367–1374.

[23] Yang DL, Jing RL, Chang XP, Li W (2007) Identification of quantitative trait loci and environmental interactions for accumulation and remobilization of water-soluble carbohydrates in wheat (*Triticum aestivum* L.) stems. Genetics 176: 571–584. 17287530 10.1534/genetics.106.068361 PMC1893045

[24] Saint Pierre C, Trethowan R, Reynolds M (2010) Stem solidness and its relationship to water-soluble carbohydrates: association with wheat yield under water deficit. Funct Plant Biol 37: 166–174.

[25] Morgan J (1980) Osmotic adjustment in the spikelets and leaves of wheat. J Exp Bot 31: 655–665.

[26] Xu X, Wang Z, Zhang J (2000) Effect of heat stress on photosynthetic characteristics of different green organs of winter wheat during grain-filling stage. Acta Bot Sin 43: 571–577.

[27] Zhang Y, Wang Z, Wu Y (2006) Stomatal characteristics of different green organs in wheat under different irrigation regimes. Acta Agron Sin 32: 70–75.

[28] Li W, Zhang B, Zhang J, Chang X, Li R, et al (2012) Exploring elite alleles for chlorophyII content of flag leaf in natural population of wheat by association analysis. Acta Agron Sin 38: 962–970.

[29] Zhang B, Shi W, Li W, Chang X, Jing R (2013) Efficacy of pyramiding elite alleles for dynamic development of plant height in common wheat. Mol Breed 32: 327–338. 23976874 10.1007/s11032-013-9873-5 PMC3748324

[30] Wang Z, Liu X, Li R, Chang X, Jing R (2011) Development of near-infrared reflectance spectroscopy models for quantitative determination of water-soluble carbohydrate content in wheat stem and glume. Anal Lett 44: 2478–2490.

[31] Yemm E, Willis A (1954) The estimation of carbohydrates in plant extracts by anthrone. Biochem J 57: 508–514. 13181867 10.1042/bj0570508 PMC1269789

[32] Somers DJ, Isaac P, Edwards K (2004) A high-density microsatellite consensus map for bread wheat (*Triticum aestivum* L.). Theor Appl Genet 109: 1105–1114. 15490101 10.1007/s00122-004-1740-7

[33] Lui K, Muse S (2005) PowerMarker: integrated analysis environment for genetic marker data. Bioinformatics 21: 2128–2129. 15705655 10.1093/bioinformatics/bti282

[34] Evanno G, Regnaut S, Goudet J (2005) Detecting the number of clusters of individuals using the software STRUCTURE: a simulation study. Mol Ecol 14: 2611–2620. 15969739 10.1111/j.1365-294X.2005.02553.x

[35] Jakobsson M, Rosenberg NA (2007) CLUMPP: a cluster matching and permutation program for dealing with label switching and multimodality in analysis of population structure. Bioinformatics 23: 1801–1806. 17485429 10.1093/bioinformatics/btm233

[36] Nei M (1972) Genetic distance between populations. Am Nat 106: 283–292.

[37] Hardy OJ, Vekemans X (2002) SPAGeDi: a versatile computer program to analyse spatial genetic structure at the individual or population levels. Mol Ecol Notes 2: 618–620.

[38] Bradbury PJ, Zhang Z, Kroon DE, Casstevens TM, Ramdoss Y, et al (2007) TASSEL: software for association mapping of complex traits in diverse samples. Bioinformatics 23: 2633–2635. 17586829 10.1093/bioinformatics/btm308

[39] Zhang Z, Ersoz E, Lai CQ, Todhunter RJ, Tiwari HK, et al (2010) Mixed linear model approach adapted for genome-wide association studies. Nat Genet 42: 355–360. 20208535 10.1038/ng.546 PMC2931336

[40] Barrero RA, Bellgard M, Zhang X (2011) Diverse approaches to achieving grain yield in wheat. Funct Integr Genomics 11: 37–48. 21221697 10.1007/s10142-010-0208-x

[41] Sayre K, Rajaram S, Fischer R (1997) Yield potential progress in short bread wheats in northwest Mexico. Crop Sci 37: 36–42.

[42] Rathinasabapathi B (2000) Metabolic engineering for stress tolerance: installing osmoprotectant synthesis pathways. Ann Bot 86: 709–716.

[43] Vereyken IJ, Chupin V, Demel RA, Smeekens S, De Kruijff B (2001) Fructans insert between the headgroups of phospholipids. Biochim Biophys Acta Biomembr 1510: 307–320. 10.1016/s0005-2736(00)00363-1 11342168

[44] Aprile A, Mastrangelo A, De Leonardis A, Galiba G, Roncaglia E, et al (2009) Transcriptional profiling in response to terminal drought stress reveals differential responses along the wheat genome. BMC Genomics 10: 279. 19552804 10.1186/1471-2164-10-279 PMC2713995

[45] Nicolas ME, Gleadow RM, Dalling MJ (1985) Effect of post-anthesis drought on cell division and starch accumulation in developing wheat grains. Ann Bot 55: 433–444.

[46] Wardlaw I, Willenbrink J (2000) Mobilization of fructan reserves and changes in enzyme activities in wheat stems correlate with water stress during kernel filling. New Phytol 148: 413–422. 10.1046/j.1469-8137.2000.00777.x 33863022

[47] Araus J, Slafer G, Reynolds M, Royo C (2002) Plant breeding and drought in C3 cereals: what should we breed for? Ann Bot 89: 925–940. 12102518 10.1093/aob/mcf049 PMC4233799

[48] Guoth A, Tari I, Galle A, Csiszar J, Pecsvaradi A, et al (2009) Comparison of the drought stress responses of tolerant and sensitive wheat cultivars during grain filling: Changes in flag leaf photosynthetic activity, ABA levels, and grain yield. J Plant Growth Regul 28: 167–176.

[49] Yang J, Huang Z, Zhu Q, Zhang J, Wang L (2000) Remobilization of carbon reserves is improved by controlled soil-drying during grain filling of wheat. Crop Sci 40: 1645–1655.

[50] Yang J, Zhang J, Wang Z, Zhu Q, Liu L (2003) Involvement of abscisic acid and cytokinins in the senescence and remobilization of carbon reserves in wheat subjected to water stress during grain filling. Plant Cell Environ 26: 1621–1631. 10.1007/s00425-002-0789-2 12172848

[51] Famoso AN, Zhao K, Clark RT, Tung C-W, Wright MH, et al (2011) Genetic architecture of aluminum tolerance in rice (*Oryza sativa*) determined through genome-wide association analysis and QTL mapping. PLoS Genet 7: e1002221. 21829395 10.1371/journal.pgen.1002221 PMC3150440

[52] Huang X, Cloutier S, Lycar L, Radovanovic N, Humphreys D, et al (2006) Molecular detection of QTLs for agronomic and quality traits in a doubled haploid population derived from two Canadian wheats (*Triticum aestivum* L.). Theor Appl Genet 113: 753–766. 16838135 10.1007/s00122-006-0346-7

[53] Marza F, Bai G, Carver B, Zhou W (2006) Quantitative trait loci for yield and related traits in the wheat population Ning7840×Clark. Theor Appl Genet 112: 688–698. 16369760 10.1007/s00122-005-0172-3

[54] Wu X, Cheng R, Xue S, Kong Z, Wan H, et al (2013) Precise mapping of a quantitative trait locus interval for spike length and grain weight in bread wheat (*Triticum aestivum* L.). Mol Breed 33: 129–138.

[55] Werner K, Friedt W, Ordon F (2005) Strategies for pyramiding resistance genes against the barley yellow mosaic virus complex (*BaMMV*, *BaYMV*, *BaYMV-2*). Mol Breed 16: 45–55.

[56] Sacco A, Di Matteo A, Lombardi N, Trotta N, Punzo B, et al (2013) Quantitative trait loci pyramiding for fruit quality traits in tomato. Mol Breed 31: 217–222. 23316114 10.1007/s11032-012-9763-2 PMC3538004

