# Supplementary figures and images for: Effects of Favorable Alleles for Water-Soluble Carbohydrates at Grain Filling on Grain Weight under Drought and Heat Stresses in Wheat
Source: PLoS One. 2014 Jul 18;9(7):e102917. doi: 10.1371/journal.pone.0102917 (PMC4103880; doi:10.1371/journal.pone.0102917)

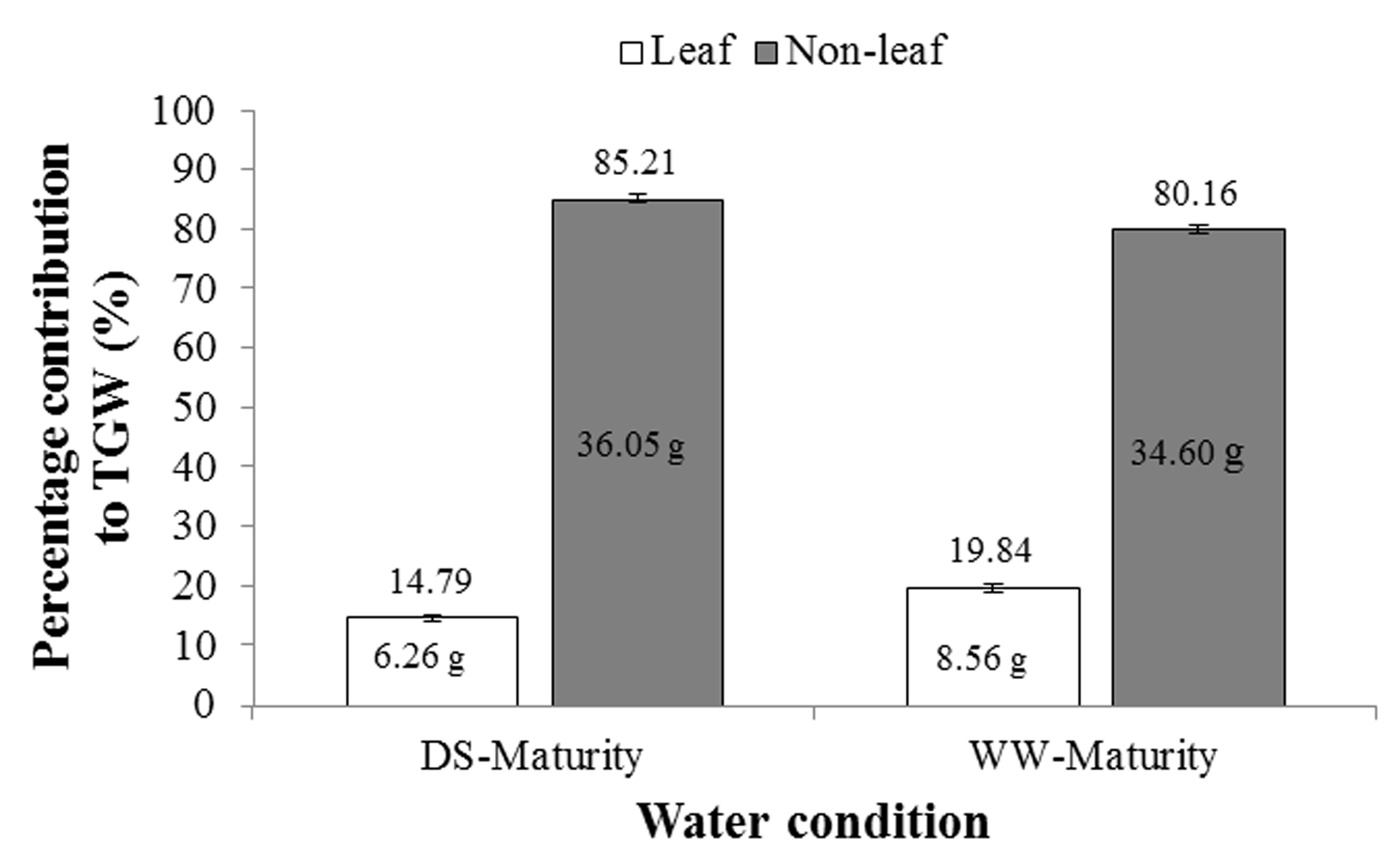

Supplement: Figure S1 — The percentage contributions of leaf and non-leaf organs to 1000-grain weight (TGW) under drought stress (DS) and well-watered (WW) conditions during grain filling. Bars indicate 2SE. The data in the columns were the absolute values of TGW (g). (TIF) [file pone.0102917.s001.tif]
